# Supplementary material for: Methodological approaches for estimating populations of the endangered dhole Cuon alpinus
Source: PeerJ. 2022 Feb 22;10:e12905. doi: 10.7717/peerj.12905 (PMC8877337; doi:10.7717/peerj.12905)
Supplement: Supplemental Information 1 [file peerj-10-12905-s001.docx]

**Supplementary file 1**

1. **SECR-Likelihood model for the leopard**

The SECR-Likelihood models were run in package *secr* in R (Version 4.1.1, R Development Core Team). Based on guidance in the package, we calculated a value for a biased estimate of spatial sigma *σ* and used a mask of suitable habitat around traps within six times this *σ* value as the buffer width. We compared the AICc scores and density plots of 3 models (Half Normal, Hazard Rate, and Negative Exponential) and selected the estimates from the best fit model.

#The detailed R code is as follows:

# Step 1: add package “secr” and set working directory

library(secr)

datadir <- system.file("extdata", package = "secr")

setwd(datadir)

#Paste data files in extdata folder in C:\Users\G\Documents\R\win-library\3.6\secr\extdata

# Step 2: make capthist object

captfile <- system.file("extdata", "lpdcapmaster.txt", package = "secr")

trapfile <- system.file("extdata", "RWStrap.txt", package = "secr")

lpdCH <- read.capthist(captfile, trapfile, detector = "proximity",

verify = TRUE)

summary(lpdCH) # Check summary

# Step 3 check biased sigma

RPSV(lpdCH, CC=TRUE)

# Step 4 Make a habitat mask by buffering around the traps

#add packages maptools and rgdal

library(maptools)

library(rgdal)

#paste shapefile in shapes folder of maptools & read shapefile into R

lpdhab <- readOGR(system.file("shapes/", package="maptools"), "bufferhablpd")

#set datum

proj4string(lpdhab) <- CRS("+init=epsg:32643")

plot(lpdhab)

clipmask<- make.mask(traps(lpdCH), type='trapbuffer', buffer=13000, poly=lpdhab)

#buffer is six times the value of biased sigma

par(mfrow =c(1,1),mar =c(1,1,1,1))

plot(clipmask,border =50,ppoly =FALSE)

plot(lpdhab,col ='lightgreen',border =NA)

plot(clipmask,dots =FALSE,mesh =grey(0.4),col =NA,polycol ='blue',add =TRUE)

plot(traps(lpdCH),detpar =list(pch =16,cex =0.8),add =TRUE)

#Step 5 Fit a null model

fit0 <- secr.fit(lpdCH, mask = clipmask, trace = FALSE)

fit0

#check buffer width

par(mar = c(4,4,1,1))

esa.plot(fit0)

abline(v=13000,lty=2,col='red')

#Fit Hazard rate model

fitHR <- secr.fit(lpdCH, mask = clipmask, detectfn='HR', trace = FALSE)

fitHR

#check buffer width

par(mar = c(4,4,1,1))

esa.plot(fitHR)

abline(v=13000,lty=2,col='red')

#Fit Negative Exponential model

fitEX <- secr.fit(lpdCH, mask = clipmask, detectfn='EX', trace = FALSE)

fitEX

#check buffer width

par(mar = c(4,4,1,1))

esa.plot(fitEX)

abline(v=13000,lty=2,col='red')

#Step 6 bundle all 3 models for comparison of estimates and AIC

fits<- secrlist(HN=fit0, HR=fitHR, EX=fitEX)

predict(fits)

AIC(fits)

#plot all 3 models to check which one is the best fit

par(mar = c(4,4,2,2))

esa.plot(fits)

abline(v=13000,lty=2,col='red')

#the model where density plateaus with increasing buffer width is the best fit

1. **SECR-Bayesian model for the leopard**

The SECR-Bayesian model was run using package SPACECAP (Gopalaswamy et al. 2012) in R. The capture history, trap deployment, and potential home range centres files were loaded to the user interface using guidance in the package, and the pixel area was set to 0.336 km^2^. Only the most general model (behavioural response) in SPACECAP was used, with the Half Normal detection function. The total number of MCMC iterations were 100,000 with a burn-in of 40,000 and thinning by 10, and data augmentation was set to 250. Default uninformative priors are a uniform Beta (1,1) prior for psi which translates into a discrete Uniform (0,M) prior on N. Here, psi is the probability that an animal in the augmented data set is a real animal in the state space, N is the number of real animals in the state space, and M is the total number of capture histories in the augmented data set. Sigma has a flat uniform [0,infty] prior, while home range centres have a uniform prior. The density plot for psi was examined for sufficiency of data augmentation, while the convergence, effective sample size, and model fit were examined to understand if the model was adequate for the data.

1. **Results of SECR models**

**Model outputs**

| SECR-Likelihood   1. N **±** SE (CI) 2. D **±** SE (CI)/100 km^2^ | 12.53 ± 4.73 (6.14 - 25.56)  3.47 ± 1.31 (1.70 - 7.08) |
| --- | --- |
| SECR-Bayesian   1. N **±** SD (CI) 2. D **±** SD (CI)/100 km^2^ | 11.84 ± 4.73 (4.26 - 20.58)  3.28 ± 1.31 (1.18 - 5.70) |

N = Abundance in study area; SE = Standard error, D = Density, SD = Standard deviation, CI = Confidence intervals
