# Supplementary material for: Methodological approaches for estimating populations of the endangered dhole Cuon alpinus
Source: PeerJ. 2022 Feb 22;10:e12905. doi: 10.7717/peerj.12905 (PMC8877337; doi:10.7717/peerj.12905)
Supplement: Supplemental Information 3 [file peerj-10-12905-s003.docx]

**Supplementary File 2**

**A. R code for site-based abundance model (Beta-Binomial/Poisson mixture model) using count data (Martin *et al.*, 2011).**

# packages

library(jagsUI)

library(mcmcOutput)

library(wiqid)

# Create model file

modeltext <- "

model{

for(i in 1:nSites){

N[i] ~ dpois(lambda)

#Beta-Binomial/Poisson mixture model: Martin et al 2011

for(j in 1:nOcc) {

C[i,j] ~ dbin(p[i], N[i])

}

p[i] ~ dbeta(A, B)T(0.01, 0.99) # Truncation (lower, upper)

}

# Priors with different a and b values [0.01,0.01; 5,3; 20,15]

lambda ~ dgamma(20, 15)

#detection of count data

mu1 ~ dunif(0, 1)

theta1 ~ dgamma(10, 2)

A <- mu1 * theta1

B <- (1 - mu1) * theta1

# derived variables p and rho

totalN <- sum(N[])

pAB <- A/(A+B) # derived detection probability

rhoAB <- 1/(A+B+1) # correlation coefficient

}"

writeLines(modeltext, con="dhole_null_model.txt")

# -----------------------------------------------------------

# Read input files

DH <- read.csv("dhole_nwg_count.csv", row.names = 1, comment = "#")

#Number of locations

nC = 27

nSites = nC

#Number of occasions

nOcc = 54

#Data

C <- DH[1:nC, 1:nOcc]

# Run JAGS

wanted <- c("pAB","rhoAB", "totalN", "lambda")

JAGSdata <- list(nSites = nSites, nOcc = nOcc, C = C)

str(JAGSdata)

Nhat <- apply(C[,1:nOcc], 1, max, na.rm=TRUE)+1

#Noc <- rep(1, nO)

#Nall <- c(Nhat, Noc)

inits <- function() list(N = Nhat , mu1 = runif(1))

# Fit data to the model

for(i in 1:100){

iserr <- tryCatch(

jagsOut <- jags(JAGSdata, inits, wanted, "dhole_null_model.txt",

n.chains=5, n.iter=10000, n.adapt=2500, n.burnin=2000, n.thin=1, DIC=TRUE, parallel=TRUE), error=function(e) e)

if(inherits(iserr, "error")){ next }

else {dump("jagsOut","Realdhole_null_model.RData")

break}

}

# Inspect output

plot(jagsOut)

#Tabulate results

mOut <- mcmcOutput(jagsOut)

summary(mOut)

res_tab<-summary(mOut)

**B. Model outputs from three scenarios (priors for N set as uninformed, partially informed, or constrained)**

**B1. Uniformed priors (gamma (0.01, 0.01))**

|  | **Mean** | **SD** | **Median** | **95% LCI** | **95% UCI** | **R-hat** |
| --- | --- | --- | --- | --- | --- | --- |
| **p** | 0.07 | 0.01 | 0.07 | 0.04 | 0.09 | 1.00 |
| **rho** | 0.10 | 0.02 | 0.10 | 0.06 | 0.14 | 1.00 |
| **N** | 189.20 | 28.26 | 187.00 | 136.00 | 244.00 | 1.00 |
| **lambda** | 7.00 | 1.16 | 6.92 | 4.85 | 9.35 | 1.00 |
| **deviance** | 568.69 | 10.38 | 568.26 | 549.07 | 589.16 | 1.00 |


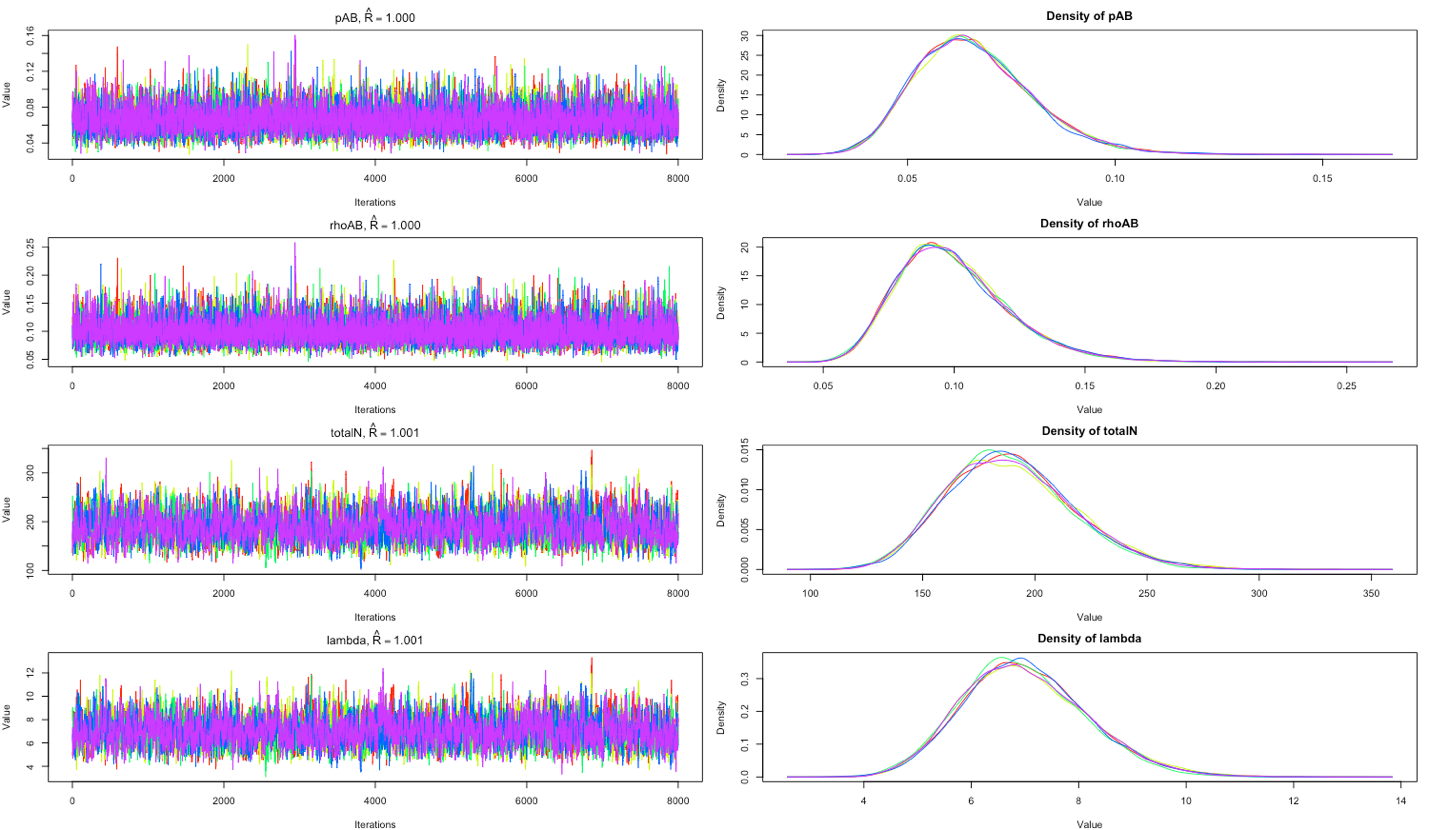


**B2. Partially informed priors (gamma (5, 3))**

|  | **Mean** | **SD** | **Median** | **95% LCI** | **95% UCI** | **R-hat** |
| --- | --- | --- | --- | --- | --- | --- |
| **p** | 0.07 | 0.02 | 0.07 | 0.04 | 0.10 | 1.00 |
| **rho** | 0.10 | 0.02 | 0.10 | 0.06 | 0.15 | 1.00 |
| **N** | 148.31 | 19.31 | 147.00 | 111.00 | 185.00 | 1.00 |
| **lambda** | 5.11 | 0.77 | 5.07 | 3.63 | 6.61 | 1.00 |
| **deviance** | 566.42 | 10.10 | 565.92 | 547.44 | 586.99 | 1.00 |


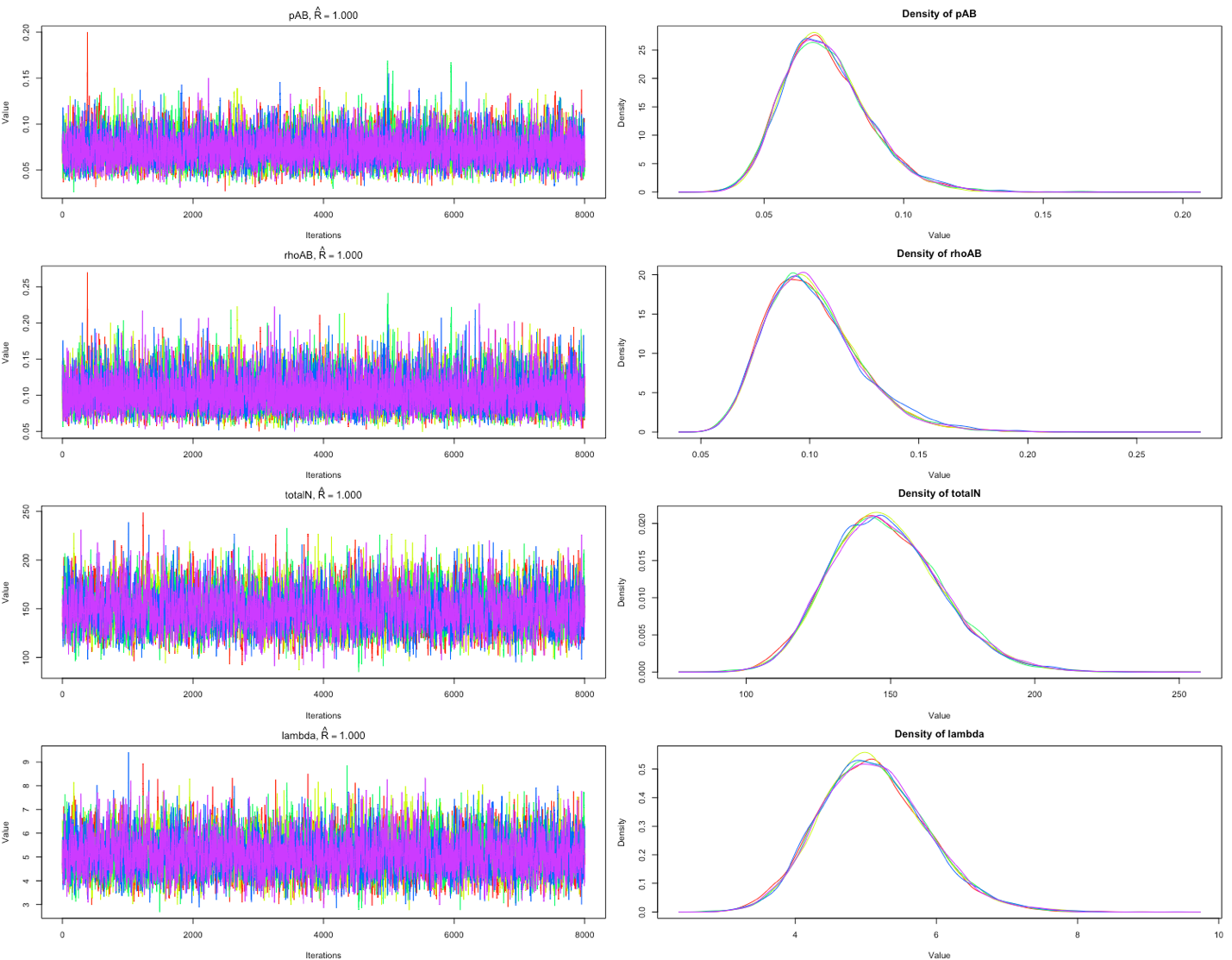


**B3. Constrained priors (gamma (20, 15))**

|  | **Mean** | **SD** | **Median** | **95% LCI** | **95% UCI** | **R-hat** |
| --- | --- | --- | --- | --- | --- | --- |
| **p** | 0.09 | 0.02 | 0.08 | 0.05 | 0.13 | 1.00 |
| **rho** | 0.11 | 0.02 | 0.10 | 0.07 | 0.15 | 1.00 |
| **N** | 104.68 | 10.17 | 104.00 | 85.00 | 124.00 | 1.00 |
| **lambda** | 2.97 | 0.36 | 2.96 | 2.28 | 3.67 | 1.00 |
| **deviance** | 564.80 | 9.75 | 564.49 | 546.22 | 584.25 | 1.00 |


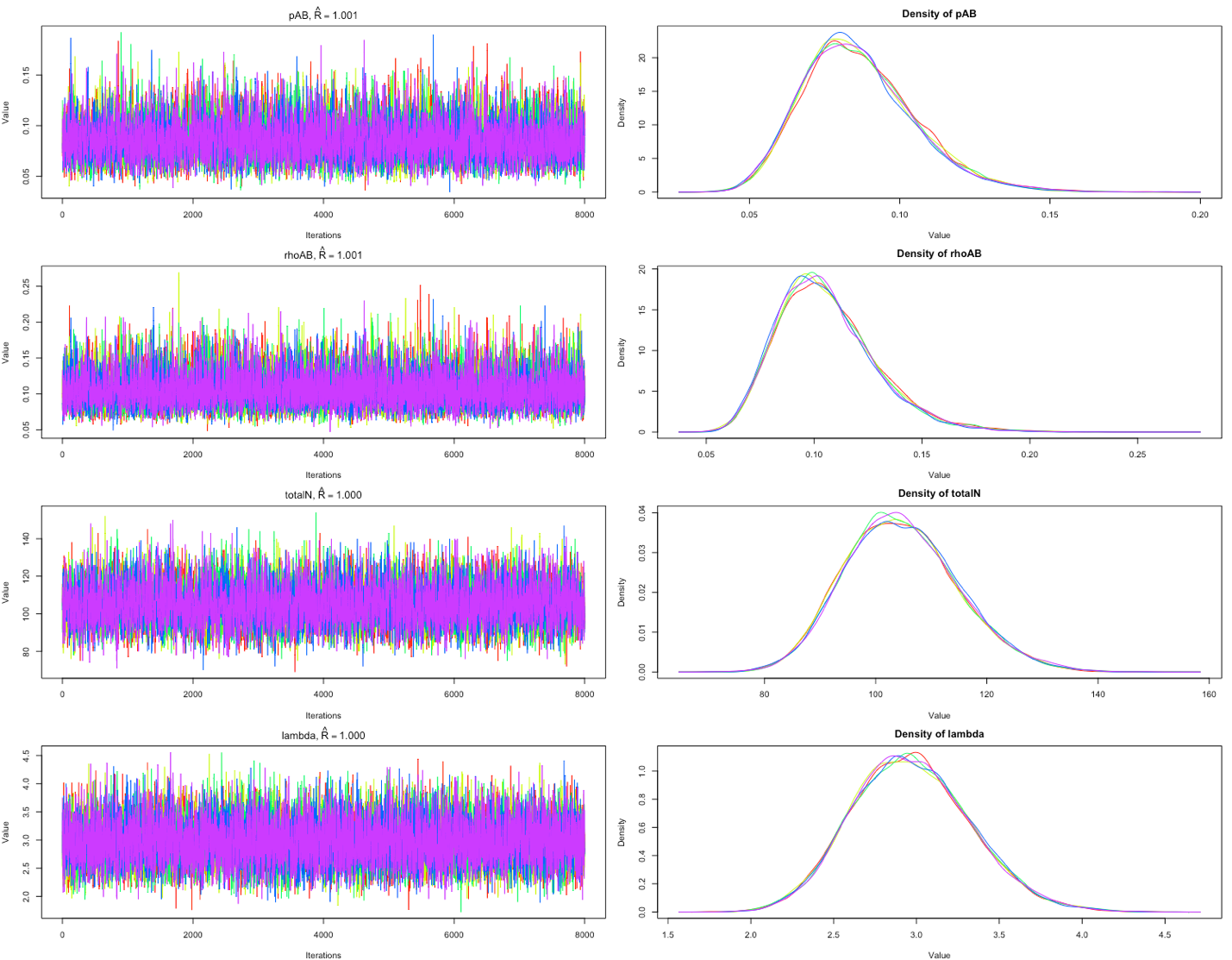


**C. R code for Space-to-Event model** **Following Moeller et al. (2018)**

devtools::install_github("annam21/spaceNtime", force = T, build_vignettes = T)

browseVignettes("spaceNtime")

library(spaceNtime)

df <- read.csv("IndiaLeoData.csv")

df <- read.csv("IndiaDholeData.csv")

df$datetime <- as.POSIXct((df$datetime), format="%Y-%m-%d %H:%M:%OS", origin="01-01-1900", tz="UTC")

df$count <- as.numeric(df$count)

df$cam <- as.numeric(df$cam)

deploy1 <- read.csv("DeploymentTime_LargestArea.csv")

deploy2 <- read.csv("DeploymentTime_LargestArea+10%.csv")

deploy3 <- read.csv("DeploymentTime_LargestArea+20%.csv")

deploy$start <- as.POSIXct((deploy$start), format="%Y-%m-%d %H:%M:%OS", origin="01-01-1900", tz="UTC")

deploy$end <- as.POSIXct((deploy$end), format="%Y-%m-%d %H:%M:%OS", origin="01-01-1900", tz="UTC")

deploy$area <- as.numeric(deploy$area)

deploy$cam <- as.numeric(deploy$cam)

study_dates <- as.POSIXct(c("2019-04-05 09:30:00", "2019-05-29 10:01:00"), tz = "GMT")

occ <- build_occ(samp_freq = 1,

samp_length = 1,

study_start = study_dates[1],

study_end = study_dates[2])

# Build encounter history

ste_eh <- ste_build_eh(df, deploy1, occ)

# Estimate abundance

#Study area 361 km2

ste_estN_fn(ste_eh, study_area = 361000000)

**D. R code for Time-to-Event model** **Following Moeller et al. (2018)**

devtools::install_github("annam21/spaceNtime", force = T, build_vignettes = T)

browseVignettes("spaceNtime")

library(spaceNtime)

df <- read.csv("IndiaLeoData.csv")

df <- read.csv("IndiaDholeData.csv")

df$datetime <- as.POSIXct((df$datetime), format="%Y-%m-%d %H:%M:%OS", origin="01-01-1900", tz="UTC")

df$count <- as.numeric(df$count)

df$cam <- as.numeric(df$cam)

deploy1 <- read.csv("DeploymentTime_LargestArea.csv")

deploy2 <- read.csv("DeploymentTime_LargestArea+10%.csv")

deploy3 <- read.csv("DeploymentTime_LargestArea+20%.csv")

deploy$start <- as.POSIXct((deploy$start), format="%Y-%m-%d %H:%M:%OS", origin="01-01-1900", tz="UTC")

deploy$end <- as.POSIXct((deploy$end), format="%Y-%m-%d %H:%M:%OS", origin="01-01-1900", tz="UTC")

deploy$area <- as.numeric(deploy$area)

deploy$cam <- as.numeric(deploy$cam)

#Dhole and leopard travel speeds from Habib *et al.*, 2021; Karanth & Sunquist, 2000; Sukmasuang et al., 2020 and Van Cleave *et al.*, 2018.

Leopard travel speeds

| Reference | Daily movement Range | Hourly movement range (lps value in model*) | Time to cover 6m camera width |
| --- | --- | --- | --- |
| Habib *et al.*, 2021 | 1862 m/day | 77.58 m/hr | 278 sec |
| Karanth & Sunquist, 2000 | 4800 m/day | 200 m/hr | 108 sec |
| Van Cleave *et al.*, 2018 | 6345 m/day | 264.37 m/hr | 81.70 sec |

Dhole travel speeds

| Reference | Daily movement Range | Hourly movement range (lps value in model*) | Time to cover 6m camera width |
| --- | --- | --- | --- |
| Habib *et al.*, 2021 | 6384 m/day | 266 m/hr | 81.20 sec |
| Sukmasuang *et al.*, 2020 | 10200 m/day | 424 m/hr | 50.94 sec |
| Sukmasuang *et al.*, 2020 | 19000 m/day | 791.7 m/hr | 27.28 sec |

*use this value in place of x where it says “per <- tte_samp_per(deploy, lps = x/3600)” in the TTE code

per <- tte_samp_per(deploy1, lps = x/3600)

# Build sampling occasions

study_dates <- as.POSIXct(c("2019-04-05 09:30:00", "2019-05-29 10:01:00"), tz = "GMT")

occ <- tte_build_occ(

per_length = per,

nper = 24,

time_btw = 2 * 3600,

study_start = study_dates[1],

study_end = study_dates[2]

)

# Build encounter history

tte_eh <- tte_build_eh(df, deploy, occ, per)

# Estimate abundance

# Study area: 361 km2

tte_estN_fn(tte_eh, study_area = 361000000)

**References**

Habib, B., Ghaskadbi, P., Khan, S., Hussain, Z., & Nigam, P. (2021). Not a cakewalk: Insights into movement of large carnivores in human‐dominated landscapes in India. Ecology and Evolution, 11(4), 1653–1666. https://doi.org/10.1002/ece3.7156

Karanth, K. U., & Sunquist, M. E. (2000). Behavioural correlates of predation by tiger (Panthera tigris), leopard (Panthera pardus) and dhole (Cuon alpinus) in Nagarahole, India. Journal of Zoology, 250(2), 255–265. https://doi.org/10.1111/j.1469-7998.2000.tb01076.x

Sukmasuang, R., Suksavate, W., Songsasen, N., Khiowsree, N., Charaspet, K., Pla-ard, M., Chanachai, Y., Thomas, W., & Srinopawan, K. (2020). Home range, movement and habitat selection of dholes (Cuon alpinus) in Khao Yai National Park, Thailand. Biodiversitas Journal of Biological Diversity, 21(12), 5915–5926. https://doi.org/10.13057/biodiv/d211257

Van Cleave, E. K., Bidner, L. R., Ford, A. T., Caillaud, D., Wilmers, C. C., & Isbell, L. A. (2018). Diel patterns of movement activity and habitat use by leopards (Panthera pardus pardus) living in a human-dominated landscape in central Kenya. Biological Conservation, 226(July), 224–237. https://doi.org/10.1016/j.biocon.2018.08.003
