# Supplementary material for: Methodological approaches for estimating populations of the endangered dhole Cuon alpinus
Source: PeerJ. 2022 Feb 22;10:e12905. doi: 10.7717/peerj.12905 (PMC8877337; doi:10.7717/peerj.12905)
Supplement: Supplemental Information 4 [file peerj-10-12905-s004.zip › Data and scripts for STE and TTE/Scripts for STE and TTE models.docx]

**Space-to-Event Model**

###Space-to-event model for dholes

#Leopard data is used to test how well the model performs compared to capture-re capture model (SCR)

#Published by Moeller et al. (2018):

#"Three novel methods to estimate abundance of unmarked animals using remote cameras"

# Follow https://github.com/keloonam/spaceNtime

devtools::install_github("annam21/spaceNtime", force = T, build_vignettes = T)

browseVignettes("spaceNtime")

library(spaceNtime)

setwd("~/OneDrive - University of Copenhagen/PhD - Dholes in Baluran/Chapters/DholeSynthesis")

#df <- read.csv("IndiaLeoData.csv")

df <- read.csv("IndiaDholeData2.csv")

#df <- IndiaDholeData

df$datetime <- as.POSIXct((df$datetime), format="%Y-%m-%d %H:%M:%OS", origin="01-01-1900", tz="UTC")

df$count <- as.numeric(df$count)

df$cam <- as.numeric(df$cam)

head(df)

#class(df$cam)

#class(df$count)

#class(df$datetime)

#deploy <- read.csv("DeploymentTime_LargestArea.csv")

#deploy <- read.csv("DeploymentTime_AverageArea.csv")

#deploy <- read.csv("DeploymentTime_LargestArea+20%.csv")

deploy <- read.csv("DeploymentTime_LargestArea+10%.csv")

#deploy <- read.csv("DeploymentTime_LargestArea-20%.csv")

#deploy <- read.csv("DeploymentTime_LargestArea-10%.csv")

deploy$start <- as.POSIXct((deploy$start), format="%Y-%m-%d %H:%M:%OS", origin="01-01-1900", tz="UTC")

deploy$end <- as.POSIXct((deploy$end), format="%Y-%m-%d %H:%M:%OS", origin="01-01-1900", tz="UTC")

deploy$area <- as.numeric(deploy$area)

deploy$cam <- as.numeric(deploy$cam)

head(deploy)

#class(deploy$start)

#class(deploy$end)

#class(deploy$area)

#class(deploy$cam)

study_dates <- as.POSIXct(c("2019-04-05 09:30:00", "2019-05-29 10:01:00"), tz = "GMT")

occ <- build_occ(samp_freq = 1, # seconds between the start of each sampling occasion !!!!!!! Check effect

samp_length = 1, # duration of each sampling occasion (seconds)

study_start = study_dates[1],

study_end = study_dates[2])

# Build encounter history

ste_eh <- ste_build_eh(df, deploy, occ)

#head(ste_eh)

# Estimate abundance

### Make sure to specify your study_area size in the same units as your camera visible areas

### Study area 361 km2

ste_estN_fn(ste_eh, study_area = 361000000)

**Time-to-Event**

###Time-to-event model for dholes

#Leopard data is used to test how well the model performs compared to capture-re capture model (SCR)

#Published by Moeller et al. (2018):

#"Three novel methods to estimate abundance of unmarked animals using remote cameras"

# Follow https://github.com/keloonam/spaceNtime

devtools::install_github("annam21/spaceNtime", force = T, build_vignettes = T)

browseVignettes("spaceNtime")

library(spaceNtime)

setwd("~/OneDrive - University of Copenhagen/PhD - Dholes in Baluran/Chapters/DholeSynthesis")

#df <- read.csv("IndiaLeoData.csv")

df <- read.csv("IndiaDholeData2.csv")

df$datetime <- as.POSIXct((df$datetime), format="%Y-%m-%d %H:%M:%OS", origin="01-01-1900", tz="UTC")

df$count <- as.numeric(df$count)

df$cam <- as.numeric(df$cam)

#head(df)

#deploy <- read.csv("DeploymentTime_LargestArea.csv")

deploy <- read.csv("DeploymentTime_LargestArea+20%.csv")

deploy$start <- as.POSIXct((deploy$start), format="%Y-%m-%d %H:%M:%OS", origin="01-01-1900", tz="UTC")

deploy$end <- as.POSIXct((deploy$end), format="%Y-%m-%d %H:%M:%OS", origin="01-01-1900", tz="UTC")

deploy$area <- as.numeric(deploy$area)

deploy$cam <- as.numeric(deploy$cam)

#head(deploy)

# Specify the length of your sampling period. This is equal to the mean amount of time (in seconds) that it takes

# for an animal to cross the average viewshed of a camera

# Estimate from Bilal's paper (Habib et al. 2020: "Not a cakewalk")

# Dhole: 259.92 m/hr

# Leopard: 99.34 m/hr

#per <- tte_samp_per(deploy, lps = 6.2)

per <- 9.2

# Build sampling occasions

study_dates <- as.POSIXct(c("2019-04-05 09:30:00", "2019-05-29 10:01:00"), tz = "GMT")

occ <- tte_build_occ(

per_length = per,

nper = 24,

time_btw = 2 * 3600,

study_start = study_dates[1],

study_end = study_dates[2]

)

# Build encounter history

tte_eh <- tte_build_eh(df, deploy, occ, per)

#head(tte_eh)

# Estimate abundance

# Study area: 361 km2

tte_estN_fn(tte_eh, study_area = 361000000)
